# Evaluation de la culture de sécurité en Etablissement d’Hébergement pour Personnes Agées Dépendantes (EHPAD): adaptation française du questionnaire Nursing Home Survey on Patient Safety Culture*

**DOI:** 10.1017/S071498081700037X

**Published:** 2017-12

**Authors:** Marie Herr, Séhéno Raharimanana, Emmanuel Bagaragaza, Philippe Aegerter, Irène Sipos, Céline Fabre, Joël Ankri, George Pisica-Donose

**Affiliations:** 1 INSERM, VIMA: Vieillissement et Maladies chroniques : approches épidémiologique et de santé publique, U1168, F-94807, Villejuif, France; 2 Univ Versailles St-Quentin-en-Yvelines, UMR-S 1168, F-78180, Montigny le Bretonneux, France; 3 AP-HP, Hôpital Sainte Périne, Département Hospitalier d’Epidémiologie et de Santé Publique, Paris, France; 4 DomusVi, Suresnes, France; 5 Pôle Recherche et Enseignement Universitaire SPES « Soins Palliatifs En Société – Ethique et Pratique Clinique », Maison Médicale Jeanne Garnier, Paris, France; 6 AP-HP, Hôpital Ambroise Paré, Unité de Recherche Clinique et Département de Santé Publique, Boulogne, France; 7 EHPAD Maison Saint-Cyr, Rennes, France

**Keywords:** aging, nursing home for the dependent elderly, patient safety culture, quality of care, questionnaire, translation, vieillissment, EHPAD, culture de sécurité, qualité des soins, questionnaire, traduction

## Abstract

The objective was to translate into French the American questionnaire “Nursing Home Survey on Patient Safety Culture” and to test the feasibility of its use in a sample of nursing homes. The questionnaire was translated by a multidisciplinary group of six experts and tested on a sample of people working in nursing homes. The questionnaire was then administered in five nursing homes. A first version of the French NHSPSC is proposed in this article. Despite similarities between items and ceiling effect for one item, the choices made were conservative to allow international comparisons. The administration of the questionnaire in five nursing homes confirmed the feasibility of the approach, with a participation of more than 50 per cent. This work made a French version of the NHSPSC available and confirmed that it is a feasible method for evaluating safety culture in nursing homes.

## RÉSUMÉ

L’objectif était d’adapter en français le questionnaire américain *Nursing Home Safety Survey on Patient Safety Culture* (NHSPSC) et tester la faisabilité de sa passation dans un échantillon d’Etablissements d’Hébergement pour Personnes Agées Dépendantes (EHPAD). Le questionnaire a été traduit par un groupe pluridisciplinaire de six experts et testé auprès d’un échantillon de personnes travaillant en EHPAD. Le questionnaire a ensuite été passé dans cinq établissements. Une première version française du NHSPSC est proposée dans cet article. Malgré l’identification de ressemblances entre items et d’un effet plafond sur un item, les choix réalisés ont été conservateurs pour permettre les comparaisons internationales. La passation du questionnaire dans cinq établissements a confirmé la faisabilité de la démarche, avec une participation basée sur le volontariat supérieure à 50 %. Ce travail propose une version française du NHSPSC et montre la faisabilité de sa passation.

## Introduction

### Prise en charge des personnes âgées dépendantes en EHPAD

Créés au début des années 2000 avec la réforme de la tarification dans les établissements pour personnes âgées, les Etablissements d’Hébergement pour Personnes Agées Dépendantes (EHPAD) représentent désormais le principal mode d’accueil des personnes âgées en France. En 2013, la Caisse Nationale de Solidarité pour l’Autonomie (CNSA) recensait 6 865 EHPAD en France, représentant une capacité d’hébergement permanent de 572 042 places (“Les soins en EHPAD en 2013: le financement de la médicalisation et le bilan des coupes Pathos”).

### Qualité des soins aux personnes âgées dépendantes

Les personnes âgées sont une population nombreuse et fragile, particulièrement exposée au risque d’événements indésirables liés aux soins du fait de la chronicité et de la fréquence de leurs comorbidités. L’amélioration de la qualité des soins aux personnes âgées est un enjeu majeur pour les EHPAD et explique le besoin d’indicateurs de sécurité des soins. Cependant, la mise en place de tels indicateurs soulève des difficultés méthodologiques. Dans un contexte de polypathologie et de soins complexes (Hillen, Vitry, & Caughey, 2015), il est plus difficile de distinguer les effets indésirables des soins des conséquences de la maladie (Barberger-Gateau, Moty, & Pérès, 2003) et un ajustement des indicateurs sur les caractéristiques des patients et de la structure peut s’avérer nécessaire (Moty, Barberger-Gateau, De Sarasqueta, Teare, & Henrard, 2003). Par ailleurs, la fréquence des troubles cognitifs complique l’évaluation de la satisfaction des résidents et la satisfaction des familles n’est que faiblement corrélée à la qualité de vie des résidents (Shippee, Henning-Smith, Gaugler, Held, & Kane, 2015).

### Culture de sécurité des soins: définition et mesure

Une approche complémentaire de la qualité des soins peut être envisagée, qui se focalise sur la culture de sécurité. Dans la triple approche de Donabedian (1988) (structures-processus-résultats), la culture de sécurité se situe parmi les indicateurs de processus. Ce n’est pas une garantie de résultat (i.e. de qualité des soins), mais un préalable important. Une étude suédoise a récemment montré que la qualité des processus prédisait largement la satisfaction des résidents en *nursing homes*, devant la qualité des structures (Kajonius & Kazemi, 2015). La *European Society for Quality in Health Care* définit la culture de sécurité comme un ensemble de comportements individuels et collectifs, fondé sur des croyances et des valeurs partagées, qui cherche continuellement à réduire les dommages aux patients. Elle est composée de plusieurs dimensions (liées au système, à la personne, à son activité et à ses interactions avec les autres personnels) (Feng, Bobay, & Weiss, 2008) qui peuvent être mesurées à l’aide de questionnaires individuels (Singla, Kitch, Weissman, & Campbell, 2006). La qualification du niveau de culture de sécurité est utilisée comme outil de pilotage d’actions d’amélioration de la qualité des soins, pour diagnostiquer les problèmes, évaluer l’efficacité d’actions correctrices puis suivre les éventuelles dérives au cours du temps (Nieva & Sorra, 2003). Les démarches d’évaluation de la culture de sécurité peuvent être considérées comme des interventions en tant que telles puisqu’elles permettent d’engager un dialogue entre professionnels d’une même structure sur la sécurité des soins. Pour ces différentes raisons, l’évaluation de la culture de sécurité des soins répond au critère 1g de la certification V2010 des établissements de santé en France (Occelli, 2010).

### Culture de sécurité : de l’hôpital aux EHPAD

Initialement inspirées du secteur aéronautique (Helmreich, 2000), les démarches d’évaluation de la culture de sécurité se développent progressivement à l’hôpital. Le questionnaire *Hospital Survey on Patient Safety Culture* (HSOPSC) développé sous l’égide de l’agence fédérale américaine *Agency for Healthcare Research and Quality* (AHRQ) a été traduit de l’anglais au français (“Questionnaire Culture Sécurité (AHRQ, traduit et validé par le Comité de Coordination de l’Evaluation Clinique et de la Qualité en Aquitaine [CCECQA]”)). La version française de ce questionnaire permet d’explorer comment les professionnels perçoivent la sécurité des soins dans leur unité et dans leur établissement de santé selon 10 dimensions de la culture de sécurité des soins explorées au travers de 40 items. En 2011, une équipe française (Occelli et al., 2011) publiait une première évaluation du niveau de culture sécurité dans 20 unités cliniques en Aquitaine (principalement des services de médecine, de psychiatrie et de chirurgie) et mettait en évidence un potentiel d’amélioration dans la réponse non punitive à l’erreur, le soutien du management pour la qualité des soins, les ressources humaines, les transferts et les transmissions.

L’évaluation de la culture de sécurité est un axe de développement de la qualité des soins encore peu utilisé en contexte gériatrique, probablement en raison du manque d’outil d’évaluation spécifique. Moyennant quelques modifications du HSOPSC, par exemple en remplaçant le terme « patient » par « résident », quelques études nord-américaines ont été réalisées dans des établissements accueillant des personnes âgées dépendantes (Castle, 2006; Castle & Sonon, 2006; Handler et al., 2006) et en long séjour gériatrique (Wagner, Capezuti, & Rice, 2009). Les résultats indiquaient notamment un potentiel d’amélioration dans quasiment toutes les dimensions de la culture de sécurité par rapport aux résultats de précédentes études en milieu hospitalier non gériatrique. Cependant, les différences de population prise en charge et d’organisation des soins entre l’hôpital et ces structures limitent les possibilités de partage d’outils d’évaluation de la culture de sécurité (Bonner, Castle, Perera, & Handler, 2008). C’est pourquoi, l’AHRQ a développé en 2007 un questionnaire d’évaluation de la culture de sécurité spécifique des *nursing homes,* c’est-à-dire des établissements accueillant des personnes âgées dépendantes. Ce questionnaire, baptisé *Nursing Home Survey on Patient Safety Culture* (NHSPSC), a été construit sur la base d’une revue de littérature (sur la sécurité des résidents en *nursing homes*, la qualité des soins, les erreurs médicales et la culture de sécurité) et d’avis d’experts. Une version préliminaire a été testée auprès de professionnels, puis la version amendée a été testée auprès des personnels de 40 établissements aux États-Unis, permettant de valider les propriétés psychométriques de l’outil (Sorra, Franklin, & Streagle, 2008). À ce jour, il n’existe pas de version française du questionnaire NHSPSC. La mise à disposition de ce questionnaire en français devrait faciliter les démarches d’évaluation de la culture de sécurité dans les établissements prenant en charge des personnes âgées dépendantes au long cours.

## Objectifs

Dans ce contexte, l’objectif principal de ce travail était d’adapter en français le questionnaire NHSPSC d’évaluation de la culture de sécurité dans les établissements accueillant des personnes âgées dépendantes (“Nursing Home Survey on Patient Safety Culture, Survey form”). L’objectif secondaire était de tester la faisabilité de l’évaluation de la culture de sécurité en EHPAD dans le cadre d’une enquête auprès d’un échantillon d’établissements.

## Méthodes

### Questionnaire NHSPSC

Le questionnaire NHSPSC s’adresse à tout le personnel des établissements accueillant des personnes âgées dépendantes. Il est composé de 42 items, regroupés en 12 dimensions de la culture de sécurité des soins (“Nursing Homes Survey on Patient Safety Culture, Items and Dimensions”) :

1. Travail en équipe (4 items)
2. Personnel et charge de travail (4 items)
3. Respect des procédures (3 items)
4. Qualification et formations (3 items)
5. Réponse non punitive à l’erreur (4 items)
6. Transferts (4 items)
7. Communication et retour d’information sur les incidents (4 items)
8. Liberté d’expression (3 items)
9. Attentes et actions des supérieurs hiérarchiques et actions pour améliorer la sécurité des résidents (3 items)
10. Perception globale de la sécurité des résidents (3 items)
11. Soutien du management pour la sécurité des résidents (3 items)
12. Amélioration continue (4 items)

Pour chaque item, la personne a la possibilité de répondre selon cinq modalités de réponse : « pas du tout d’accord / pas d’accord / ni pour ni contre / d’accord / tout à fait d’accord » ou « jamais / rarement / de temps en temps / la plupart du temps / toujours » selon la formulation de l’item.

Chaque dimension de la culture de sécurité donne lieu à un score, qui s’exprime en pourcentage moyen d’avis favorables. Prenons l’exemple de la première dimension, « travail en équipe ». Celle-ci est composée de quatre items et, pour chaque item, on peut calculer un pourcentage d’avis favorables correspondant aux deux modalités de réponse allant dans le sens d’une culture de sécurité développée. Pour l’item 1 (« Dans l’établissement, chacun considère les autres avec respect. »), le pourcentage d’avis favorables correspond à la somme du nombre de réponses « d’accord » et « tout à fait d’accord » sur le nombre total des réponses. On notera que les avis favorables peuvent correspondre aux réponses « pas du tout d’accord » ou « pas d’accord » dès lors que la formulation de l’item va à l’encontre d’une culture de sécurité développée (par exemple : « Pour gagner du temps, il arrive que le personnel ne respecte pas ou fasse l’impasse sur les protocoles »). La moyenne du pourcentage d’avis favorables sur les quatre items de la dimension 1 donne le score de la dimension « travail en équipe » (si les pourcentages d’avis favorables aux quatre items sont 61 %, 50 %, 69 %, 54 %, alors le score de la dimension est de 59 %). Par convention, une dimension est dite “à améliorer” si son score est inférieur à 50 % et “développée” s’il est supérieur à 75 % (J. Sorra et al., 2008). Un score global de culture sécurité peut être calculé en faisant la moyenne des scores des 12 dimensions (Thomas et al., 2012).

À ces 42 items s’ajoutent deux questions finales sur l’évaluation globale du niveau de sécurité dans l’établissement et la propension des personnels interrogés à recommander l’établissement.

Enfin, la dernière partie du questionnaire s’intéresse aux caractéristiques du répondant, en termes de profession, d’ancienneté dans l’établissement, de nombre d’heures travaillées chaque semaine, de travail de nuit, de travail auprès de malades atteints de la maladie d’Alzheimer, etc.

### Protocole d’adaptation transculturelle

Après avoir au préalable obtenu l’accord explicite de l’AHRQ pour réaliser l’adaptation transculturelle du questionnaire NHSPSC en français, un protocole en trois étapes a été mis en œuvre selon les indications de l’AHRQ (“Translation Guidelines for the AHRQ Surveys on Patient Safety Culture”) :

#### Étape 1: Constitution d’un groupe de traduction

Le groupe de traduction était composé de six personnes travaillant dans le domaine de la santé publique, de la gériatrie et de la gérontologie, dont deux médecins de santé publique, un épidémiologiste, un directeur d’EHPAD, un doctorant en qualité des soins et un étudiant de Master 2. L’animation du groupe a été réalisée par un binôme de traduction (épidémiologiste-étudiant de Master 2).

#### Étape 2: Préparation d’une version préliminaire du questionnaire

Une première traduction du questionnaire a été proposée par le binôme de traduction. Ce travail s’appuyait sur 1/ la version française du questionnaire hospitalier HSOPSC pour les items communs (“Questionnaire Culture Sécurité (AHRQ, traduit et validé par le CCECQA)”) et 2/ sur les documents mis à disposition par l’AHRQ expliquant le sens de chaque item du questionnaire afin d’en faciliter la traduction (“Nursing Home Survey on Patient Safety Culture, Background and Information for Translators”). Cette version préliminaire du questionnaire a été examinée par l’ensemble des membres du groupe de traduction, avec une attention particulière portée sur le sens et la simplicité des questions. Les propositions de changement ont été intégrées au questionnaire par le binôme de traduction et validées par l’ensemble des membres du groupe.

#### Étape 3: Test du questionnaire en focus groups

Pour vérifier la clarté des items et leur bonne compréhension par le public cible, deux entretiens collectifs (*focus groups*) avec des personnes travaillant en EHPAD ont été organisés dans un même établissement (privé commercial avec une capacité d’accueil de 106 lits). Le premier *focus group* a réuni sept personnes, de profession infirmier diplômé d’état coordinateur (IDEC) (*n* = 1), infirmier diplômé d’état (IDE) (*n* = 1), aide-soignant (AS) (*n* = 3), responsable hôtelier (*n* = 1) et responsable hébergement (*n* = 1). Le second *focus group* a réuni cinq autres personnes, de profession IDE (*n* = 1), AS (*n* = 2), chef cuisinier (*n* = 1) et aide médico-psychologique (AMP) (*n* = 1). Chaque entretien s’est déroulé de la même façon, avec un temps dédié au remplissage du questionnaire et un temps de discussion autour de chaque item du questionnaire. Chaque séance a duré une heure et demie. À noter une participation plus active des IDE et IDEC lors des *focus groups* par rapport aux AS. Le binôme de traduction a opéré les modifications nécessaires au vu des remarques des personnes travaillant en EHPAD et a fait valider ces modifications par l’ensemble du groupe.

### Enquête auprès de cinq établissements

La passation du questionnaire a été réalisée dans cinq EHPAD d’Ile-de-France (privés commerciaux) afin de tester la faisabilité de la démarche, en particulier la participation, le temps et la qualité du remplissage (pourcentage de non-réponse par item), ainsi que les éventuels effets plancher et plafond du questionnaire. Au cours d’une période d’enquête d’une à deux semaines, toute personne travaillant dans l’établissement (personnel de jour et de nuit, libéral ou salarié) était invitée à remplir le questionnaire. L’enquête était annoncée sur site par des affichages et l’information était relayée auprès du personnel de chaque établissement par les directeurs et IDEC. Chaque questionnaire était accompagné d’une enveloppe retour à déposer dans une urne prévue à cet effet afin de préserver la confidentialité des informations recueillies. Les modalités pratiques de l’enquête (calendrier, mode de distribution des questionnaires, place de l’urne retour) ont été précisées avec la direction de chaque établissement. Les questionnaires ont été distribués au personnel selon trois modalités : mise à disposition des questionnaires au niveau du poste d’accueil, distribution pendant les temps de transmissions ou distribution individuelle (par l’IDEC ou avec le bulletin de salaire).

Les données de l’enquête ont été saisies à l’aide du logiciel Epidata et analysées avec le logiciel Stata® v13.

## Résultats

### Travail du groupe de traduction pour proposer une version préliminaire du questionnaire

Premièrement, la formulation des modalités de réponse aux questions a été revue par rapport au HSOPSC, en remplaçant la modalité intermédiaire « neutre » par « ni pour ni contre ». En revanche, la formulation des modalités de réponse aux questions fréquentielles est restée fidèle au questionnaire hospitalier. Concernant la formulation des 42 questions sur les différentes dimensions de la culture de sécurité, la proposition initiale du binôme de traduction a été retenue d’emblée pour 14 d’entre elles. Les discussions engagées par le groupe de traduction sur les autres questions portaient sur le choix des termes, sur le choix des exemples, sur des questions d’interprétation ou sur la possibilité de simplifier/clarifier les questions.

Parmi les termes discutés, on peut citer les exemples suivants (en dernier la formulation retenue) :

- *« To be part of a team »* : faire partie de l’équipe de travail / d’une équipe de travail / d’une équipe

- *« To get really busy »* : avoir trop de travail / être débordé / avoir beaucoup de travail

- *« To be blamed »* : être visé / être rendu responsable

- *« Resident’s care plan »* : prise en charge / plan de soins

- *« Staff ideas and suggestions are valued »* : les idées et les suggestions du personnel sont bien accueillies / bien accueillies et écoutées / prises en considération

L’apport des médecins et gériatres du groupe de traduction a été essentiel, en particulier pour le choix des exemples. Par exemple :

- Question originale du NHSPSC : « *Staff follow standard procedures to care for residents. »*

- Indication de traduction : « *Staff follow the same set of established procedures each time they perform a task—examples of tasks include putting in a catheter, dressing a wound, feeding residents who have had a stroke and have difficulty swallowing, and using a lift. »*

- Adaptation française : Le personnel suit des protocoles standards pour la prise en charge des résidents (par exemple pour les pansements, l’alimentation des personnes ayant des troubles de la déglutition ou l’utilisation du lève-malade).

Pour certains items, un certain degré d’interprétation a été nécessaire. Dans l’exemple suivant, nous avons considéré que comprendre une formation impliquait de la mettre en pratique :

- Question originale du NHSPSC : « *Staff understand the training they get in this nursing home. »*

- Indication de traduction : « *Staff not only receive training in the nursing home but also understand what they are supposed to learn. »*

- Adaptation française : Le personnel comprend et met en pratique les formations suivies dans cet établissement.

Dans certains cas, il était plus aisé de se baser sur les indications de traduction plutôt que sur la question originale pour réaliser l’adaptation française. Par exemple :

- Question originale du NHSPSC : « *Staff are treated fairly when they make mistakes. »*

- Indication de traduction : « *Staff expect supervisors to investigate all factors, including systems reasons, to determine why mistakes happen instead of immediately deciding that only the staff member is at fault. Staff are not treated harshly when a human error is made. »*

- Adaptation française : Avant de mettre en cause le personnel, les erreurs sont analysées pour en comprendre les causes.

La proximité de certains items a été pointée du doigt, notamment sur la formation, le respect des procédures et le dialogue avec la hiérarchie. Par exemple (dans leur version originale) :

- « *Staff use shortcuts to get their work done faster.* » et « *To make work easier, staff often ignore procedures.* »

- « *Management asks staff how the nursing home can improve resident safety.* » et « *Management listens to staff ideas and suggestions to improve resident safety.* »

Concernant la liste des professions en EHPAD, celle-ci a été revue en fonction de la pratique française. À la question sur le temps de travail dans l’établissement, les modalités de réponse ont été adaptées au régime français des 35 h (découpage de la durée de travail hebdomadaire en moins de 15 h / 16 à 24 h / 25 à 34 h / 35 h et plus au lieu de moins de 15 h / 16 à 24 h /25 à 40 h / plus de 40 h).

### Enseignements des focus groups

Le terme culture de sécurité des soins a fait débat, pour deux raisons. La première est que l’on ne sait pas forcément d’emblée ce que recouvre l’expression « culture de sécurité ». C’est pourquoi les quelques phrases introductives du questionnaire ont été précisées, en utilisant les termes suggérés par les participants eux-mêmes, tels que « organisation du travail » et « communication avec les autres professionnels de l’établissement ». Le deuxième point de discussion portait sur le terme « soins ». L’IDEC a souligné le fait qu’un EHPAD était un lieu de vie avant d’être un lieu de soins. Cette remarque, approuvée par les autres membres du groupe, a conduit à simplifier l’intitulé du questionnaire, qui mentionne désormais la culture de sécurité en EHPAD plutôt que la culture de sécurité des soins en EHPAD.

Aucun item du questionnaire n’a posé de réel problème de compréhension. En revanche, quelques demandes de précision ont été émises, en particulier à la question 9 sur le terme « direction » (direction de l’établissement ou direction du groupe en cas d’appartenance à une enseigne) et à la question 10 sur la nature des problèmes envisagés chez les résidents (physique ou autre). En outre, la question 3, relative à la capacité à faire face à la charge de travail, a été précisée en ajoutant le terme « actuellement », pour considérer les conditions de travail présentes et effectives.

Certains exemples ont paru trop liés aux soins pour des personnels non soignants, par exemple pour le cuisinier. Néanmoins, nous n’avons pas modifié ces exemples, car il semblait difficile de proposer des exemples en rapport avec chacun des métiers représentés au sein d’un EHPAD sans alourdir considérablement le questionnaire.

Dans la partie « informations générales » du questionnaire existait une question interrogeant le répondant sur le caractère direct ou indirect de ses contacts avec les résidents. Cette question a été jugée peu pertinente par l’ensemble des participants. À la différence des structures hospitalières, les EHPAD sont des structures de plus petite taille dans lesquelles rares sont les personnes qui ne côtoient pas les résidents. Cette question a donc été supprimée et l’espace libéré a été utilisé pour poser une question supplémentaire sur l’ancienneté dans le métier (et pas seulement dans la structure). À noter que ces questions ne contribuent pas au calcul du score de culture de sécurité.

Également dans la partie « informations générales », les entretiens ont fait ressortir le fait que si l’on précisait son métier avec le degré de finesse du questionnaire original (dans la partie du questionnaire s’intéressant aux profils des répondants), la confidentialité des réponses pouvait être remise en question. Les métiers ont donc été regroupés par catégories : personnel de direction, personnel administratif, médecin, infirmier, aide-soignant, autre personnel de soins, personnel hôtelier, de restauration et d’animation, stagiaire et autre.

### Elaboration d’une version française du NHSPSC

Au final, le processus d’adaptation transculturelle a permis d’aboutir à la version proposée en annexe. Les choix réalisés ont été relativement conservateurs, de façon à ne pas restreindre les possibilités de comparaisons internationales.

### Enquête auprès de cinq établissements

Parmi les 243 personnes travaillant dans les cinq établissements enquêtés, 126 ont participé à l’enquête, soit un taux de participation de 52 %. Selon l’établissement, ce taux variait entre 44 % et 64 %. Les AS et les autres personnels de soins étaient les plus représentés (respectivement *n* = 34 et *n* = 24) (Tableau 1). Seuls trois directeurs et un médecin coordonnateur ont répondu. Une meilleure participation a été relevée dans les établissements où les questionnaires étaient distribués et remplis pendant les temps de transmission. La durée d’enquête, initialement prévue à une semaine a dû être prolongée à deux semaines pour optimiser la participation, sauf pour un établissement.

**Tableau 1 :** Distribution des répondants à l’enquête par type d’emploi

Type d’emploi	N (%)
Personnel de direction	3 (2,6)
Personnel administratif	3 (2,6)
Médecin	1 (0,9)
Infirmier	16 (14,0)
Aide-soignant	34 (29,8)
Stagiaire	11 (9,7)
Autre personnel de soins*	24 (21,2)
Personnel hôtelier, d’animation et de restauration	16 (14,0)
Autre	6 (5,3)

* Kinésithérapeute, ergothérapeute, psychomotricien, psychologue, diététicien, pédicure/podologue, aide médico-psychologique, personnel de soins en gérontologie, pharmacien

La durée médiane de remplissage du questionnaire était de 14 minutes (min = 2 ; max = 40 minutes). Le pourcentage moyen de non-réponse par item était de 4,6 %, avec un maximum de non-réponse pour le temps de remplissage (26,2 %) et les informations générales. Si l’on ne considère que le cœur du questionnaire, i.e. les questions portant sur la culture de sécurité, le pourcentage de non-réponse descend à 3,4 % (min = 0,8 % ; max = 7,1 %), avec une tendance à plus de non-réponses dans les derniers items du questionnaire.

Les résultats de cette enquête ont révélé un effet plafond, c’est-à-dire un pourcentage d’avis favorables supérieur ou égal à 80 %, pour quatre items :

- Le personnel suit des protocoles standards pour la prise en charge des résidents.

- Le personnel alerte s’il voit quelque chose qui pourrait mettre en danger un résident.

- Mon supérieur hiérarchique se soucie des problèmes de sécurité des résidents.

- Cet établissement est un lieu sûr pour les résidents.

Cependant, seul le second item faisait l’objet d’avis favorables à plus de 80 % dans les cinq établissements, les autres items ayant conservé une certaine variabilité entre établissements.

Un effet plancher, c’est-à-dire un pourcentage d’avis favorables inférieur à 20 %, a été retrouvé pour un unique item :

- Le personnel est obligé de faire les choses trop rapidement, car la charge de travail est trop importante.

Là encore, cet effet plancher ne résistait pas à l’analyse stratifiée par établissement puisque seuls deux établissements sur cinq avaient effectivement un pourcentage d’avis favorables inférieur à 20 % à cet item.

Concernant les résultats à proprement parler, la dimension 10, « perception globale de la sécurité des résidents », est apparue comme très développée avec un score de 77 % d’avis favorables. En revanche, les dimensions 2 et 5, « personnel et charge de travail » et « réponse non punitive à l’erreur », étaient les moins développées avec des scores d’environ 38 % (Tableau 2). Le score global de culture de sécurité était de 59 %, avec des scores par établissement compris entre 46 % à 73 % (Tableau 3). Le score global de culture de sécurité variait selon la profession : 90 % chez l’unique médecin participant, 89 % parmi les personnels de direction (*n* = 3), 70 % parmi le personnel infirmier (*n* = 16), 62 % parmi les personnels paramédicaux (*n* = 24), 54 % parmi les AS (*n* = 34) et 53 % pour le personnel hôtelier, de restauration et d’animation (*n* = 16).

**Tableau 2 :** Scores de culture de sécurité dans chacune des 12 dimensions de la version française du NHSPSC calculés dans un échantillon de cinq EHPAD d’Ile-de-France

Dimension	Pourcentage d’avis en faveur d’une culture de sécurité développée (%)
1. Travail en équipe (4 items)	58,5
2. Personnel et charge de travail (4 items)	37,7
3. Respect des procédures (3 items)	50,8
4. Qualification et formations (3 items)	64,9
5. Réponse non punitive à l’erreur (4 items)	38,4
6. Transferts (4 items)	64,3
7. Communication et retour d’information sur les incidents (4 items)	70,2
8. Liberté d’expression (3 items)	53,8
9. Attentes et actions des supérieurs hiérarchiques et actions pour améliorer la sécurité des résidents (3 items)	68,9
10. Perception globale de la sécurité des résidents (3 items)	77,0
11. Soutien du management pour la sécurité des résidents (3 items)	59,6
12. Amélioration continue (4 items)	63,8

**Tableau 3 :** Score global de culture de sécurité dans chacun des cinq EHPAD d’Ile-de-France enquêté

EHPAD	Nombre de répondants	Score global de culture de sécurité (%)
#1	30	61,7
#2	27	46,3
#3	31	63,9
#4	17	49,1
#5	21	72,9

## Discussion

### Principaux résultats

Ce travail a mobilisé un groupe de traduction pluridisciplinaire pour traduire et adapter en français le questionnaire international NHSPSC. Il s’agit du premier questionnaire français d’évaluation de la culture de sécurité en EHPAD. Ce questionnaire a été testé selon deux modalités : lors de *focus groups* impliquant des représentants de différents métiers, et lors d’une étude pilote dans cinq établissements volontaires. Ce pilote confirme la faisabilité de ce type d’enquête en EHPAD, avec un taux de réponse moyen dépassant 50 %.

### Discussion du choix de l’outil et de la méthode

Premièrement, le choix du NHSPSC comme outil d’évaluation de la culture de sécurité a été motivé par plusieurs raisons : 1/ il s’agit d’un des rares outils spécifiquement conçus pour évaluer la culture de sécurité en *nursing homes*, 2/ il s’agit d’un outil validé pour lequel il existe une banque de données de référence alimentée par les résultats d’enquêtes menées dans différents pays et 3/ ce choix s’inscrit dans la continuité des travaux français sur le HSOPSC. Le champ couvert par les *nursing homes* américains n’est cependant pas parfaitement superposable à celui des EHPAD français : les *nursing homes* américains étant susceptibles de mettre en œuvre des soins médicaux relevant de l’hôpital en France (Sanford et al., 2015). À noter que le NHSPSC est le questionnaire le plus employé au niveau international, bien que d’autres questionnaires existent, ciblant souvent un type de métier (IDE ou AS notamment) (Bonner et al., 2008).

Le choix de la méthode d’adaptation transculturelle s’est porté sur une traduction par un groupe d’experts plutôt que sur une méthode par rétro-traduction (méthode qui consiste à traduire le questionnaire une première fois en français et retraduire ce questionnaire dans sa langue originale ; les différences entre la version originale et la version retraduite permettent d’identifier les situations problématiques). La possibilité de réunir dans un même groupe un large panel de compétences, en santé publique, en gérontologie, en qualité des soins et en direction d’EHPAD, offrait, selon nous, une richesse d’information supérieure à la méthode de rétro-traduction. Passé cette première étape, le test du questionnaire auprès de professionnels dans le cadre de *focus groups* a permis de préciser la formulation du questionnaire pour qu’il soit le plus compréhensible possible.

Bien que l’enquête réalisée dans cinq établissements nous ait permis d’apporter des éléments concrets sur la faisabilité de ce type d’évaluation en EHPAD, la taille d’échantillon ne nous permettait pas de répliquer l’étude des propriétés psychométriques du questionnaire (un minimum de 500 observations est requis pour étudier un questionnaire comprenant plus de 40 items), comme cela a pu être fait pour les versions originale et suisse (Zúñiga, Schwappach, De Geest, & Schwendimann, 2013).

### Enseignements de l’enquête auprès de cinq établissements

La relativement bonne participation à l’enquête (supérieure à 50 %, qui est le seuil critique en deçà duquel les résultats sont jugés peu représentatifs selon l’AHRQ) montre l’acceptabilité de la démarche pour les personnes travaillant en EHPAD. D’après notre expérience, la participation est favorisée par la distribution directe des questionnaires aux personnels, par rapport à la mise à disposition des questionnaires « en libre-service ». Bien qu’efficace, on peut cependant craindre que cette méthode ne soit pas la plus propice à instaurer un climat de confidentialité. De plus, la distribution lors des transmissions exclut les personnels non concernés par les transmissions, notamment le personnel hôtelier, le personnel de direction ainsi que le personnel administratif. De façon générale, il est important de définir des modalités de retour des questionnaires permettant de garantir la confidentialité des réponses, en accord avec la direction et l’IDEC dont la collaboration et l’adhésion à la démarche sont essentielles à la réussite de l’enquête. Il peut également être utile de prévoir un délai supplémentaire pour permettre de relancer les personnels et améliorer une participation insuffisante.

Bien que notre échantillon d’étude n’était pas représentatif du panorama des EHPAD français (puisqu’il s’agissait de cinq EHPAD privés commerciaux), les dimensions prioritaires en termes d’amélioration de la culture de sécurité retrouvées dans cette étude, à savoir « réponse non punitive à l’erreur » et « personnel et charge de travail » étaient les mêmes que celles identifiées à partir de la banque de données de l’AHRQ portant sur 263 équivalent-EHPAD (Sorra et al., 2014). Il convient de souligner que ces résultats reflètent principalement les opinions des personnels les plus nombreux, en l’occurrence les AS et autres paramédicaux. Or, les résultats suggèrent une forte variabilité des niveaux de culture de sécurité selon la profession, avec de meilleurs niveaux parmi le personnel dirigeant et les soignants par rapport aux auxiliaires. Ces différences s’expliquent probablement par des différences de formation initiale, des différences de risque selon les métiers et des différences en termes de responsabilité. Elles posent la question de la sensibilisation à la culture de sécurité dans le cadre de la formation continue pour les personnels peu qualifiés intervenant auprès de personnes âgées vulnérables. Par ailleurs, l’interprétation des résultats doit tenir compte du caractère déclaratif des réponses, avec un risque de biais de déclaration malgré le souci de confidentialité des réponses.

### Place de la culture de sécurité dans la démarche qualité en EHPAD

Les démarches en faveur du développement de la culture de sécurité s’inscrivent souvent dans un cadre réglementaire, notamment la certification dans le cas des établissements de santé ou l’accréditation dans le cas d’un laboratoire de biologie médicale. Dans le cas des EHPAD, la qualité des soins est évaluée au moyen d’évaluations internes (au moins tous les cinq ans) et d’évaluations externes organisées par l’Agence Nationale de l’Évaluation et de la Qualité des Établissements et Services Sociaux et Médico-Sociaux (ANESM). Les champs des évaluations externe et interne sont les mêmes afin d’assurer la complémentarité des analyses portées sur un même établissement ou service et de fait, être en mesure d’apprécier les évolutions et les effets des mesures prises pour l’amélioration continue du service rendu. L’engagement dans une démarche qualité, le périmètre de celle-ci (enquêtes de satisfaction, évaluations de pratiques, etc.) et son pilotage (groupes travail, communication autour de la qualité, suivi) sont des points qui figurent dans la grille ANGELIQUE (Application Nationale pour Guider une Évaluation Labellisée Interne de Qualité pour les Usagers des Établissements). L’évaluation de la culture de sécurité peut aider les établissements à établir leur bilan initial et préciser les actions d’amélioration de la qualité prioritaires, et ainsi contribuer à faciliter leurs démarches d’évaluation.

### Quel bénéfice tangible attendre d’une amélioration de la culture de sécurité ?

Une étude menée aux États-Unis auprès d’infirmiers de 81 unités de soins de 42 établissements de santé de court séjour a montré que plus le score de culture de sécurité était élevé, moins il y avait d’erreurs médicamenteuses et d’infections urinaires dans l’unité (Hofmann & Mark, 2006). Le niveau de culture de sécurité des soins pourrait également être lié à la santé des personnels soignants, avec un niveau élevé réduisant le risque de douleurs dorsales (Hofmann & Mark, 2006) et d’épuisement professionnel au sein du personnel infirmier (Spence Laschinger & Leiter, 2006). Les relations entre culture de sécurité et qualité effective des soins sont très peu documentées en contexte gériatrique et, à notre connaissance, il n’existe qu’une seule étude, américaine, ayant montré une relation significative entre une culture de sécurité développée et un moindre risque de contention et de chute (Thomas et al., 2012). Bien que non significatifs, ces résultats se retrouvent à l’état de tendance dans l’étude suisse de Zuniga et al. (Zúñiga et al., 2013).

## Conclusion

Ce travail propose une méthode simple et faisable d’évaluation de la culture de sécurité en EHPAD, à l’aide d’un auto-questionnaire validé dans sa version originale et traduit en français. Ce type de démarche peut aider les établissements prenant en charge des personnes âgées dépendantes à réaliser un premier diagnostic et à mettre en place des actions d’amélioration de la qualité.
